# Nueva variante en el gen *COL4A3*: etiología de un síndrome de Alport tipo 2 en varón de 38 años con sospecha de nefritis hereditaria

**DOI:** 10.1515/almed-2021-0027

**Published:** 2021-07-21

**Authors:** Paula Sienes Bailo, José Luis Bancalero Flores, Raquel Lahoz Alonso, María Santamaría González, Alex Gutiérrez Dalmau, Sara Álvarez de Andrés, Silvia Izquierdo Álvarez

**Affiliations:** Servicio de Bioquímica Clínica, Hospital Universitario Miguel Servet, Zaragoza, España; Servicio de Nefrología, Hospital Universitario Miguel Servet, Zaragoza, España; NIMGenetics, Madrid, España

**Keywords:** *COL4A3*, hematuria glomerular familiar, síndrome de Alport

## Abstract

**Objetivos:**

Los pacientes con síndrome de Alport experimentan una pérdida progresiva de la función renal, pérdida auditiva neurosensorial y anomalías oculares. Está causado por mutaciones en los genes *COL4A5* (herencia ligada al cromosoma X), *COL4A3* y *COL4A4* (herencia autosómica dominante o recesiva), que codifican respectivamente las cadenas α3, α4 y α5 del colágeno tipo IV. En ausencia de tratamiento, el cuadro clínico progresa desde hematuria microscópica hacia proteinuria, insuficiencia renal progresiva y enfermedad renal terminal. En la actualidad, el trasplante renal supone el único tratamiento eficaz. Las pruebas genéticas de secuenciación masiva son el método de elección para el diagnóstico de esta patología.

**Presentación del caso:**

Se presenta el caso de un varón joven con enfermedad renal crónica que fue finalmente trasplantado, en el que el estudio genético permitió conocer la etiología de su contexto clínico, un síndrome de Alport tipo 2 de herencia autosómica recesiva. Se detectó que el paciente era portador de dos variantes de cambio de sentido en heterocigosis compuesta (configuración *trans*) en *COL4A3*: una probablemente patogénica c.4981C>T (p.Arg1661Cys) en el exón 52 heredada vía materna previamente descrita y otra de significado clínico incierto c.943G>A (p.Gly315Ser) en el exón 17 heredada vía paterna que no había sido reportada anteriormente en la literatura ni en las bases de datos consultadas.

**Conclusiones:**

La confirmación desde el punto de vista genético permitió asesorar adecuadamente al paciente y familiares directos.

## Introducción

El síndrome de Alport (SA) es una enfermedad rara que se caracteriza por la presencia de anomalías estructurales y disfunción en la membrana basal glomerular (MBG), así como en las membranas basales de otros órganos como el ojo y el oído. Los pacientes con SA experimentan una pérdida progresiva de la función renal con cambios ultraestructurales típicos en la MBG, pérdida auditiva neurosensorial y anomalías oculares [1, 2]. Este síndrome está causado por mutaciones en los genes *COL4A3*, *COL4A4* y *COL4A5*, que codifican respectivamente las cadenas α3, α4 y α5 del colágeno tipo IV, presentes en la MBG y en otras membranas basales. Según el gen mutado, se han descrito tres patrones de herencia para este síndrome: el 80% de los casos se transmiten mediante herencia ligada al cromosoma X (SALX o SA tipo 1, MIM#301050) asociándose con mutaciones en *COL4A5*, el 15% se hereda de forma autosómica recesiva (SAAR o SA tipo 2, MIM#203780) y el 5% restante, de forma autosómica dominante (SAAD o SA tipo 3, MIM#104200). Tanto el SAAR como el SAAD están causados por alteraciones en *COL4A3* o *COL4A4* [3, 4]. En el caso del SAAR, la falta de expresión de la red α345(IV) de colágeno en las membranas basales se asocia con un fenotipo severo de aparición temprana que afecta por igual a ambos sexos. El riesgo de enfermedad renal terminal (ERT) de estos pacientes es del 100%, con una tasa de progresión y edad de inicio de las manifestaciones extrarrenales influenciadas por el genotipo [5].

En ausencia de tratamiento, el cuadro clínico progresa desde hematuria microscópica hacia proteinuria, insuficiencia renal progresiva y ERT. En la actualidad, el trasplante renal supone el único tratamiento eficaz, si bien pueden beneficiarse del tratamiento precoz con nefroprotectores, como los inhibidores de la enzima convertidora de angiotensina (IECA) o los antagonistas de los receptores de angiotensina (ARA), frenando la progresión a ERT y mejorando la esperanza de vida [6]. Por ello, un diagnóstico temprano de afectos y familiares en riesgo es crucial. Actualmente, las pruebas genéticas son el método de elección por ser menos invasivas que las biopsias de piel o riñón y poseer una especificidad diagnóstica del 95%. La secuenciación Sanger ha sido la prueba estándar, aunque en los últimos años las técnicas de secuenciación masiva (NGS) han demostrado ser alternativas muy prometedoras [7, 8].

Presentamos el caso de un varón joven con enfermedad renal crónica (ERC) que fue finalmente trasplantado, en el que el estudio genético permitió conocer la etiología de su contexto clínico, un síndrome de Alport tipo 2 de herencia autosómica recesiva, con el hallazgo de una nueva variante en el gen *COL4A3* no documentada hasta la fecha. La confirmación desde el punto de vista genético permitió asesorar adecuadamente al paciente y familiares directos.

## Caso clínico

Varón de 38 años remitido desde Nefrología en julio de 2019 para estudio genético de alteraciones en los genes del colágeno. El paciente, afecto de ERC supuestamente atribuible a enfermedad de la membrana basal, fue trasplantado en 2014 y posteriormente desarrolló hipoacusia. Sin antecedentes familiares de interés, hijo único con progenitores no consanguíneos.

Como antecedentes personales destacar que a los 3 años fue diagnosticado de una infección urinaria, sin clínica y con poca fiebre. Con 4 años se le diagnosticaron 2 procesos de infección del tracto urinario con microhematuria y febrícula. A los 5 años presentó amigdalitis de repetición y algún episodio miccional con microhematuria y a partir de los 6, hematurias macroscópicas recurrentes. En los sedimentos urinarios se observaron cilindros granulosos y/o granuloso-hemáticos, con elevado porcentaje de hematíes dismórficos, por lo que se realizó una biopsia renal siendo diagnosticado de glomerulopatía proliferativa mesangial difusa. Siguieron persistiendo las hematurias recurrentes, junto con proteinurias en rango nefrótico, manteniendo la función renal normal (Tabla 1).

**Tabla 1:** Evolución de los parámetros de laboratorio relacionados con la función renal del paciente, antes y después del trasplante (2014).

	Intervalo de referencia	Pre-trasplante										Post-trasplante	
		2006	2007	2008	2009	2010	2011	2012	2013	2014	2014	Mes 12 Nov/2015	Año 6 Nov/2020
**Suero**													
Creatinina	0,67–1,17 mg/dL	1,00	1,30	1,21	1,19	1,52	1,57	1,58	3,74	4,79	6,76	2,10	2,18
Ácido úrico	3,5–7,2 mg/dL	7,8	8,9	8,3	7,6	5,7	5,4	4,4	8,7	8,1	10,9	7,50	4,81
FGe (MDRD)	>60 mL/min/1,73 m^2^	–	–	71,93	72,79	54,49	52,13	51,41	18,90	14,11	9,48	36,33	35,01
Proteínas totales	6,6–8,3 g/dL	6,2	6,4	5,3	5,7	6,2	5,6	5,6	6,8	5,9	6,3	7,3	7,2
Albúmina	3,5-5,2 g/dL	3,9	4,1	3,0	3,1	3,7	3,5	3,6	4,2	3,6	4,0	4,9	4,8
Potasio	3,5-5,1 mEq/L	4,5	4,8	4,9	4,2	5,2	5,1	5,2	6,9	5,6	7,4	4,4	4,0
Calcio	8,6–10 mg/dL	9,5	9,7	9,6	9,3	10,1	9,4	9,4	10,2	9,5	9,6	10,1	10,2
Fósforo	2,5–4,50 mg/dL	3,8	3,5	3,7	4,2	4,2	4,7	4,0	4,1	5,3	4,2	3,30	3,50
Colesterol	120–220 mg/dL	217	229	305	194	210	183	180	221	173	146	167	196
**Orina**													
Proteínas totales	<0,03 g/L	–	0,05	–	–	–	–	–	1,62	3,56	6,34	0,10	0,00
Proteínas totales (24 horas)	<0,1 g/24 h	2,18	–	2,30	1,56	3,84	5,48	7,18	–	–	–	–	–
Hematíes	0-5 por campo visual	10–20	5–10	40–50	60–80	40–50	10–20	40–50	–	–	–	10–25	3–5

FGe, filtrado glomerular estimado; MDRD, Modification of Diet in Renal Disease.

Estudios radiológicos posteriores reflejaron ecográficamente hallazgos propios de nefropatía, destacando un bazo megálico con significación de vasos de retorno, con imagen adenomatosa/hiperplásica. A nivel renal se observó hiperdensidad cortical renal bilateral. A los 29 años el paciente mostró progresiva reducción del filtrado glomerular y proteinuria a pesar del tratamiento, realizándose una nueva biopsia renal con ampliación para estudio ultraestructural, describiéndose un riñón con glomérulos y membrana basal adelgazada e imágenes de *splitting,* concordante con nefritis hereditaria. La evolución posterior evidenció una ERC evolutiva (Tabla 1) y hallazgos ecográficos congruentes con ese diagnóstico (riñones de morfología conservada y disminución de la diferenciación corticomedular). Ante la progresión de la ERC del paciente, y el ofrecimiento de su padre como donante, de 65 años y sin signos clínicos de nefropatía, en 2014 se procedió a un trasplante renal anticipado de donante vivo emparentado observándose mejoría en la función renal (Tabla 1).

En 2020, tras solicitud del nefrólogo, se realizó en una muestra de ADN extraído de sangre periférica con EDTA un exoma dirigido mediante NGS (NIMGenetics, Madrid, Spain) para estudio de posibles variantes en 10 genes asociados con síndrome de Alport y otros síndromes para diagnóstico diferencial (*BSND, COL4A1, COL4A3, COL4A4, COL4A5, COL4A6, GATA3, MYH9, NPHS1* y *NPHS2).* Las variantes identificadas se cotejaron con la información recogida en bases de datos específicas sobre otras variantes descritas en asociación a un fenotipo conocido (HGMD, ClinVar) y en bases de datos de frecuencias poblacionales (dbSNP, Genome Aggregation Database (gnomAD), 1000 Genome Project, NHLBI-ESP 6500 exomes). Asimismo, se estimó la patogenicidad de las variantes identificadas usando ocho sistemas de predicción incluidos en el paquete ANNOVAR (SIFT, PolyPhen2, LRT, MutationTaster, MutationAssessor, FATHMM, MetaSVM y CONDEL) y dos adicionales (PROVEAN, Align GVGD) para mutaciones de cambio de sentido. Para establecer el grado de conservación de las variantes entre especies, se realizó un alineamiento de secuencias que comparó 9 secuencias aminoacídicas informadas para las cadenas α3 del colágeno tipo IV de diferentes especies.

En el análisis genético y posterior estudio de segregación en los progenitores, se detectó que el paciente era portador de dos variantes de cambio de sentido en heterocigosis compuesta (configuración *trans*) en *COL4A3*: una probablemente patogénica c.4981C>T (p.Arg1661Cys) en el exón 52 heredada vía materna previamente descrita y otra de significado clínico incierto c.943G>A (p.Gly315Ser) en el exón 17 heredada vía paterna que no había sido reportada anteriormente en la literatura ni en las bases de datos consultadas (Tabla 2). La variante c.4981C>T (p.Arg1661Cys) estaba registrada en la base de datos HGMD (CM014049) en asociación con SA. Asimismo, había sido previamente incluida en la base de datos ClinVar (Variation ID: 287915) con conflicto de interpretación entre variante patogénica, probablemente patogénica y de significado incierto. La variante c.943G>A (p.Gly315Ser) no había sido registrada en asociación con ningún fenotipo específico.

**Tabla 2:** Variantes del gen *COL4A3* detectadas en el caso índice y en sus progenitores.

	Gen	Nomenclatura variante	Exón	Cigosidad	Efecto	Categorización variante	Herencia	Fenotipo
A	*COL4A3*	c.943G>A p.(Gly315Ser)	17	Het	Missense	VSI	AR	Síndrome de Alport tipo 2
	*COL4A3*	c.4981C>T p.(Arg1661Cys)	52	Het	Missense	VPP	AR	Síndrome de Alport tipo 2
B	*COL4A3*	c.943G>A p.(Gly315Ser)	17	Het	Missense	VSI	AR	Asintomático
C	*COL4A3*	c.4981C>T p.(Arg1661Cys)	52	Het	Missense	VPP	AR	Asintomática

Het, heterocigosis; VSI, variante de significado incierto; VPP, variante probablemente patogénica; AR, autosómica recesiva.Variantes genómicas identificadas en: (A) estudio de secuenciación masiva del exoma humano en sangre periférica (exoma clínico) incluyendo 10 genes asociados a síndrome de Alport y síndromes incluidos en el diagnóstico diferencial: *BSND, COL4A1, COL4A3, COL4A4, COL4A5, COL4A6, GATA3, MYH9, NPHS1* y *NPHS2* (ExoNIM^®^ NIMGenetics). (B–C) evaluación del patrón de segregación de las variantes descritas en el paciente mediante secuenciación Sanger: (B) en el padre y (C) en la madre (NIMGenetics, Madrid, España).

## Discusión

Identificamos una nueva variante en *COL4A3* en combinación con una variante descrita previamente en heterocigosis compuesta. Mutaciones en el gen *COL4A3* se asocian al SA tipo 3 y a la hematuria familiar benigna (MIM#141200) con herencia autosómica dominante; y, con herencia autosómica recesiva, al SA tipo 2. La presentación clínica del caso descrito (proteinuria severa progresiva, hipoacusia y ERT temprana) sería más compatible con SA tipo 2. La importancia de realizar un análisis genético molecular tras los primeros síntomas para el diagnóstico precoz y elección de la alternativa terapéutica más apropiada queda demostrada en este estudio.

**Figura 1:**
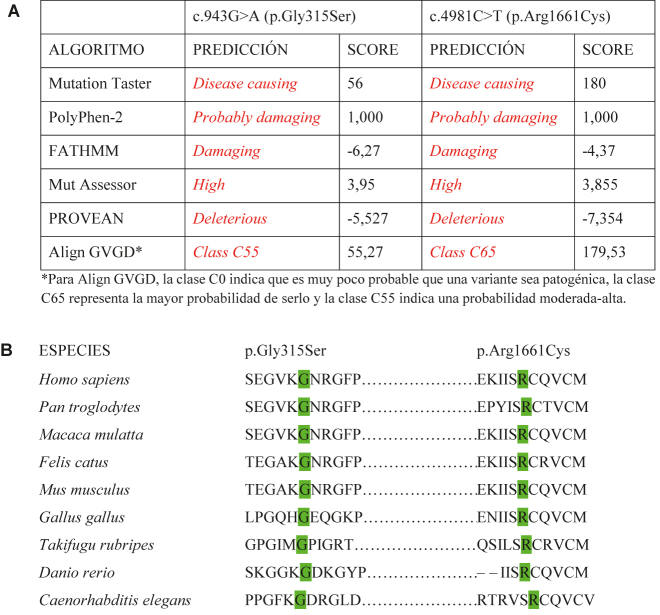
(A) Impacto funcional de las variantes c.943G>A (p.Gly315Ser) y c.4981C>T (p.Arg1661Cys) y (B) análisis de conservación entre especies de los aminoácidos implicados. (A) Resultados devueltos por varios predictores *in silico* (MutationTaster, PolyPhen2, FATHMM, MutationAssessor, PROVEAN y Align GVGD) en el análisis de patogenicidad de las dos variantes halladas en el caso índice. Estas herramientas predicen el posible impacto de las sustituciones aminoacídicas en la estructura y función de la α3(IV). (B) Alineamiento de secuencias de aminoácidos parciales de la α3(IV) de nueve especies diferentes. Se comprueba que ambas posiciones (315 y 1661) están altamente conservadas (9/9) en la evolución.

**Figura 2:**
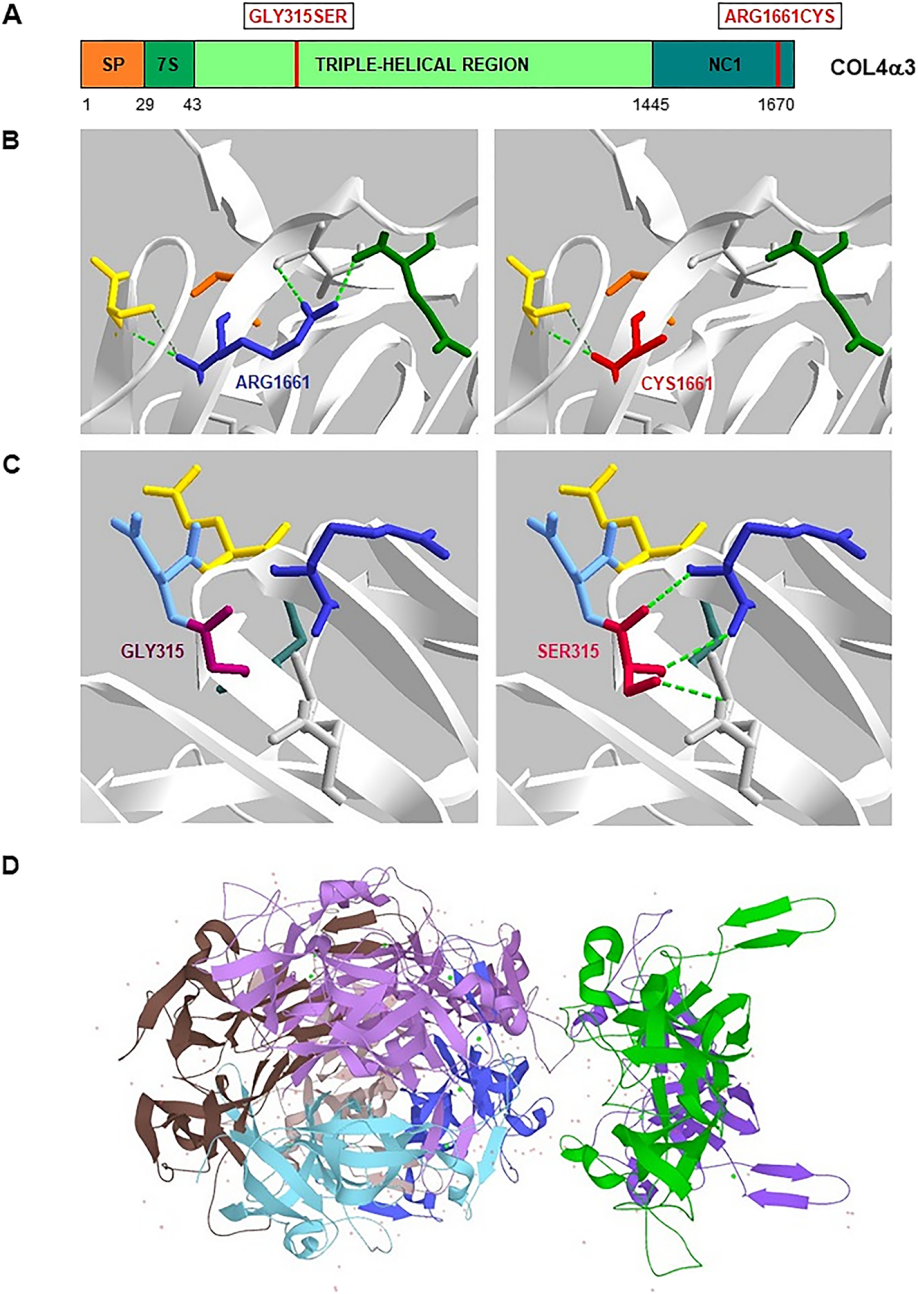
Dominios de la cadena alfa-3 de colágeno tipo IV, α3(IV), y representación tridimensional de las variantes c.943G>A (p.Gly315Ser) y c.4981C>T (p.Arg1661Cys). (A) Las cadenas alfa-3 del colágeno tipo IV tienen un dominio no colágeno globular rico en cisteínas en el extremo C-terminal (NC1), interrupciones frecuentes de las repeticiones GXY en el dominio largo central de triple hélice que aportan flexibilidad a la triple hélice y un dominio de triple hélice corto (7S) en la región N-terminal. En el esquema se indica la localización de las variantes descritas: la variante p.Gly315Ser se encuentra en el dominio de triple hélice central, mientras que la variante p.Arg1661Cys se encuentra en el dominio NC1. (B–C) Representación 3D de las variantes en la α3(IV) (PDB 5NB0, SPDBviewer). En B se muestra como la sustitución de la Arg1661, aminoácido con carga positiva, por una Cys, aminoácido polar sin carga y de menor tamaño, altera el patrón de interacciones de tipo puentes de hidrógeno, interacciones hidrofóbicas, electrostáticas y contactos de Van der Waals que se establecen con aminoácidos cercanos afectando finalmente a la estabilización de la estructura tridimensional de la α3(IV) y a su funcionalidad. De forma análoga se muestra en C la sustitución de la Gly315, aminoácido polar sin carga, por una Ser, aminoácido polar sin carga de mayor tamaño, que establece nuevos puentes de hidrógeno ausentes en la proteína nativa. (D) Representación tridimensional general del octámero de α3(IV) (UniprotKB).

La variante c.4981C>T (p.Arg1661Cys) presente en el dominio no colágeno C-terminal de la cadena alfa-3 de colágeno tipo IV (NC1) afecta a una posición altamente conservada en las seis cadenas de colágeno tipo IV y altera la estabilidad del protómero y conformación del dominio NC1 donde se cree que la nueva cisteína forma enlaces disulfuro dispares [9, 10] (Figuras 1 y 2). Esta variante, ha sido identificada previamente en otros pacientes con SAAR, tanto en homocigosis [10] como en heterocigosis compuesta [10], [11], [12], diagnosticados a una edad más temprana (10–18 años) si bien existe un caso más tardío (44 años). Como nuestro caso, estos pacientes presentaron adelgazamiento de la MBG y proteinuria en rango nefrótico; uno, además, pérdida auditiva y ninguno enfermedad ocular [12]. Junto a esta, la variante c.943G>A (p.Gly315Ser) se asume causante del cuadro clínico del paciente ya que segrega dentro de la familia, afecta a un aminoácido altamente conservado de la región de triple hélice de la α3(IV) y se prevé probablemente perjudicial y causante de enfermedad en la mayoría de predictores consultados (Figura 1). Además, la presencia de glicinas siguiendo el patrón Gly-X-Y-Gly-X-Y, es importante para la organización espacial de la triple hélice de colágeno [9, 12] (Figura 2). El curso más rápido de ERT y la aparición más temprana (<20 años) de otros pacientes que presentan la variante p.Arg1661Cys en heterocigosis compuesta, podría deberse a la menor patogenicidad de la variante p.Gly315Ser frente a sustituciones de Gly que afectan a otras posiciones (p.ej. p.Gly631Val o p.Gly830Asp) [10, 11].

La presencia de mutaciones heterocigotas en *COL4A3* se asocia con un espectro de fenotipos que van desde la ausencia de sintomatología hasta la hematuria aislada asintomática o incluso cuadros de ERC, sordera neurosensorial y anomalías oculares, aun en la misma familia [5, 9]. En nuestro caso, el diagnóstico genético previo al trasplante habría contribuido a un mejor asesoramiento de los riesgos asociados a la donación del padre, dado su estado de portador de la variante de significado incierto, aunque actualmente, con 72 años, presenta función renal conservada, sin albuminuria ni signos de progresión a ERC. La madre, portadora de la variante probablemente patogénica, también continua asintomática, pese a que esta variante ha sido reportada en una ocasión en asociación con ERT en estado de heterocigosis [13].

El estudio genético de su descendencia y otros familiares en riesgo está indicado, así como su vigilancia periódica prestando especial atención a la aparición de hipertensión, proteinuria, hematuria o insuficiencia renal. En caso de identificar las variantes heterocigotas en *COL4A3* podría valorarse el inicio de terapia con IECA o ARA para frenar precozmente la progresión a ERT [14]. Así, la importancia de las pruebas genéticas no debe obviarse en el diagnóstico de enfermedades renales como el SA ya que un diagnóstico genético preciso puede conducir al inicio temprano del tratamiento, a la prevención del empeoramiento rápido de la enfermedad y a la correcta selección de parientes donantes para trasplante o planificación familiar [15].

## Puntos de aprendizaje

– La presencia de una nueva variante combinada con una variante descrita previamente en *COL4A3* en estado heterocigótico compuesto es compatible con un SAAR.
– Conocer la causa molecular de la enfermedad renal nos permite mejorar la precisión diagnóstica, estimar el riesgo de recurrencia en las familias, ofrecer un asesoramiento genético adecuado y elegir la mejor opción terapéutica evitando acciones ineficaces.
– Las pruebas genéticas en el SA también permiten excluir la enfermedad en algunos familiares, lo que les permite ser considerados como posibles donantes renales.
